# Stroke Care Within the COVID-19 Pandemic—Increasing Awareness of Transient and Mild Stroke Symptoms Needed

**DOI:** 10.3389/fneur.2020.581394

**Published:** 2020-10-09

**Authors:** Timo Uphaus, Sonja Gröschel, Eyad Hayani, Marianne Hahn, Falk Steffen, Klaus Gröschel

**Affiliations:** Department of Neurology, University Medical Center of the Johannes Gutenberg University Mainz, Mainz, Germany

**Keywords:** cerebral ischemia, cerebral infarct, transient ischemic attack, COVID-19, mechanical thrombectomy

## Abstract

**Background:** The COVID-19 pandemic might affect health care resources and alter patient admission to hospital in case of stroke or transient ischemic attack (TIA). We aim to determine whether the COVID-19 pandemic is affecting utilization of recanalization procedures and numbers of patients with stroke and TIA admitted to a primary care stroke center.

**Methods:** In this retrospective observational study, we compared patients admitted from January 2019 until February 2020 with patients admitted during the COVID-19 pandemic (March/April 2020) in Germany. We included patients with stroke (hemorrhagic or ischemic) or TIA as classified by International Statistical Classification of Diseases and Related Health Problems version 10 (ICD-10).

**Results:** The number of patients per month with ischemic stroke or TIA was found to have significantly decreased from January 2019 until February 2020 compared to the COVID-19 pandemic (March/April 2020) (ischemic stroke 69.1 ± 4.5 vs. 55 ± 5.7, *p* < 0.001, TIA 22.1 ± 4.1 vs. 14.5 ± 6.4, *p* < 0.034). Contrarily, percentages and numbers of recanalization procedures per month were not influenced by the COVID-19 pandemic (intravenous thrombolysis [iv-tPA] 9.4 ± 3.7 vs. 10.5 ± 0.5, *p* = 0.697, mechanical thrombectomy [MT] 13.1 ± 3.1 vs. 14.5 ± 3.5, *p* = 0.580, iv-TPA or MT 19.4 ± 4.1 vs. 19.0 ± 0.0, *p* = 0.889).

**Conclusions:** During the COVID-19 pandemic, resources of the healthcare system in a primary care university hospital in Germany still allowed for unchanged numbers of recanalization procedures due to ischemic stroke. However, the numbers of patients admitted to the hospital specifically due to ischemic stroke or TIA decreased, suggesting that the awareness for non-disabling stroke symptoms has to be increased.

## Introduction

On March 11, 2020, the Corona Virus Disease 2019 (COVID-19) was declared a global pandemic by the World Health Organization (1). Due to the predominantly pulmonary manifestation of COVID-19, it requires long and resource-consuming intensive care treatment (2) and resulted in a shortage of healthcare system capacities in southwest Europe (3). Therefore, by mid-March 2020, when COVID-19 cases exponentially increased in Germany, people were told to stay at home and avoid close contact with individuals outside their inner family in order to flatten the infection curve and thereby avoid similar shortages of intensive care capacities as in neighboring countries. Moreover, the organizational structure of hospitals changed in order to be prepared for an increased admission of COVID-19 patients by reducing non-emergency ambulatory patients and elective hospital admissions. These circumstances led to the question of what happens with other severe disease such as stroke, especially considering reports from Italian colleagues depicting almost a disappearance of patients with ischemic strokes within their hospitals (3) and similar reports from North America (4, 5) and Brazil (6). We aim to elucidate whether the COVID-19 pandemic affected either the admission of patients to our primary care stroke center or the rates of stroke recanalization therapies.

## Materials and Methods

### Study Design and Participants

For the current analysis, we used data from patients admitted between January 2019 and April 2020 to our primary care university hospital in Mainz, Germany. Patients were selected by principal diagnosis of ischemic/hemorrhagic stroke or transient ischemic attack (TIA), as classified by the International Statistical Classification of Disease and related health problems−10th revision (ICD-10) used for financial reimbursement from the healthcare providers. Additionally, the acute stroke treatment was classified into no recanalization therapy vs. recanalization therapy (intravenous thrombolysis [iv-tPA] or mechanical thrombectomy [MT]) using procedural codes. We then divided the patient cohort into patients admitted from January 2019 until February 2020 and those admitted in March/April 2020. We performed an additional hand-search of all patients admitted to the stroke unit within March/April 2020 to identify patients who were not yet ICD-diagnosis/procedural coded. In line with regional legislation of the Ethics Committee of the Landesärztekammer Rheinland-Pfalz (Landeskrankenhausgesetz § 36 und § 37) due to the retrospective nature of the current analysis no ethical approval or informed consent to participate was deemed necessary.

### Statistical Analysis

Data is presented as median (Interquartile Range [IQR]), mean (±standard deviation [SD]), or numbers with percentages, if not indicated otherwise. For univariate analysis Student's *t*-test, Mann-Whitney *U*-test, and Chi-square tests were used. Number of strokes, TIA, and recanalization procedures per months between January 2019 and February 2020 were compared to number of strokes in March/April 2020 by unpaired *t*-test. A significant difference was considered for *p* < 0.05. Statistical analysis was performed using SPSS (version 23, IBM, Armonk, NY, USA).

## Results

**Figure 1:**
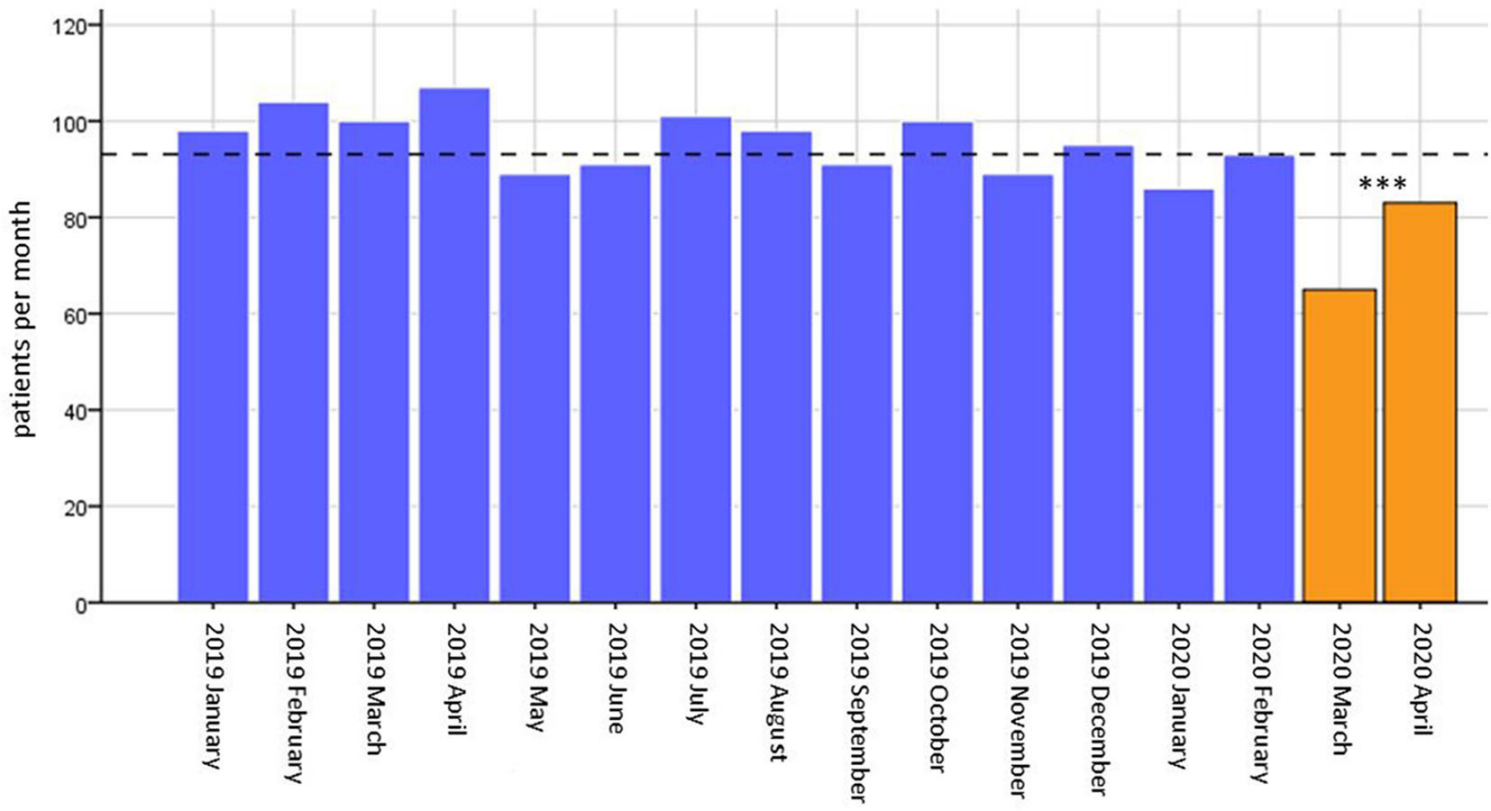
Number of patients admitted per month. Absolute numbers of patients admitted per month plotted as bars. The mean of patients admitted per month is displayed as a dashed line (93.1 ± 10.0 patients per months). *** = *p* < 0.001 for difference between patients per month from January 2019 until February 2020 compared to March/April 2020.

**Figure 2:**
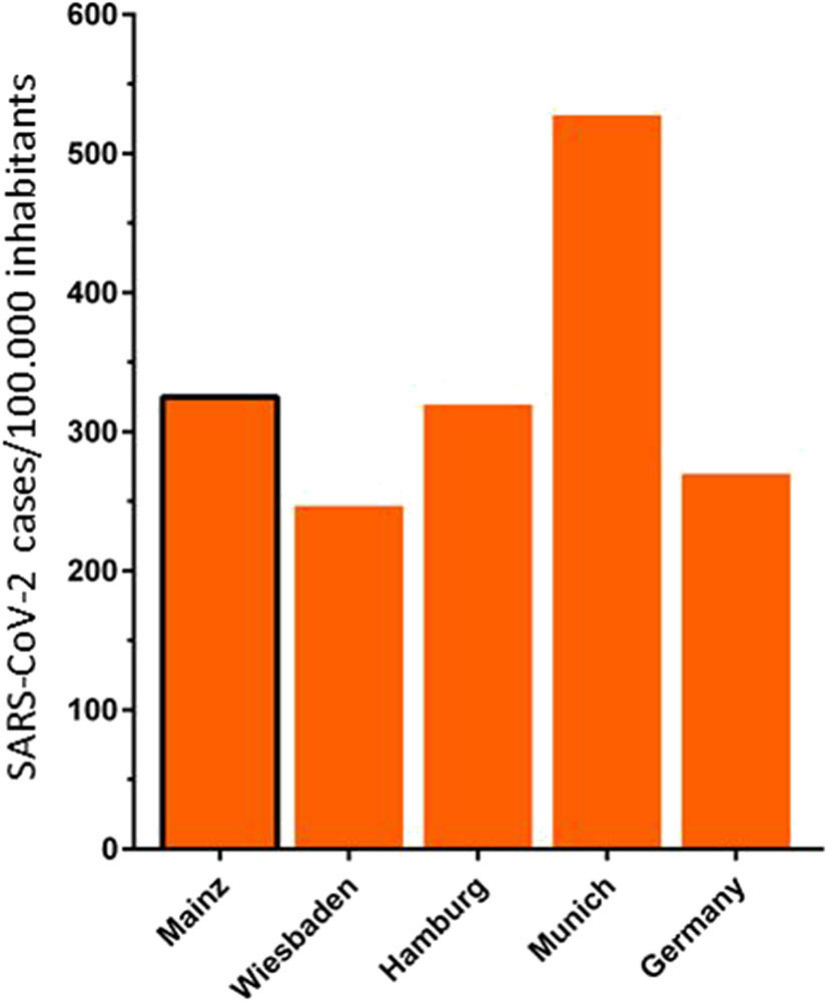
Incidence of confirmed SARS-CoV-2 cases in Mainz, compared to other urban areas and Germany. Mainz 272 SARS-CoV-2 cases/100.000 inhabitants, Wiesbaden 249 SARS-CoV-2 cases/100,000 inhabitants, Hamburg 322 SARS-CoV-2 cases/100,000 inhabitants, Munich 530 SARS-CoV-2 cases/100,000 inhabitants, Germany 272 SARS-CoV-2 cases/100,000 inhabitants (7, 8).

In total, 1,490 stroke/TIA patients (mean age 72.6 ± 13.3 years, 46.4% female) were admitted to our primary care hospital during the study period, yielding 93.1 ± 10.0 patients per month (see Figure 1). In March/April 2020, there was a decrease in patients admitted due to ischemic/hemorrhagic stroke or TIA (74 ± 12.7) as compared to the preceding 14 months with an average of 95.9 ± 6.3 patients per month (*p* < 0.001) (see Figure 1). Patient characteristics such as age (72.9 ± 12.5 vs. 72.6 ± 13.4; *p* = 0.777), female sex (53.4 vs. 45.7%; *p* = 0.075), and days of hospitalization (7.8 ± 6.8 vs. 8.2 ± 7.1; *p* = 0.583) were equally distributed between the two observation periods. In order to get an impression of the severe acute respiratory syndrome coronavirus 2 (SARS-CoV-2) situation in the location of the center examined within this report, we compared the incidence of confirmed SARS-CoV-2 cases in Mainz with other urban areas and Germany as a whole. To calculate the incidence we accessed the number of inhabitants in a central registry (7) and the confirmed SARS-CoV-2 cases at the Robert-Koch-Institut (8) on August 18. With regard to the incidence of SARS-CoV-2 cases, Mainz (325 cases/100,000 inhabitants) is in the range of other urban areas, e.g., Wiesbaden (249 cases/100,000 inhabitants) and Hamburg (322 cases/100,000 inhabitants) and Germany-wide (272 cases/100,000 inhabitants). However, there are parts of Germany that are more severely affected by SARS-CoV-2, e.g., Munich with 530 cases/100,000 inhabitants (see Figure 2).

**Figure 3:**
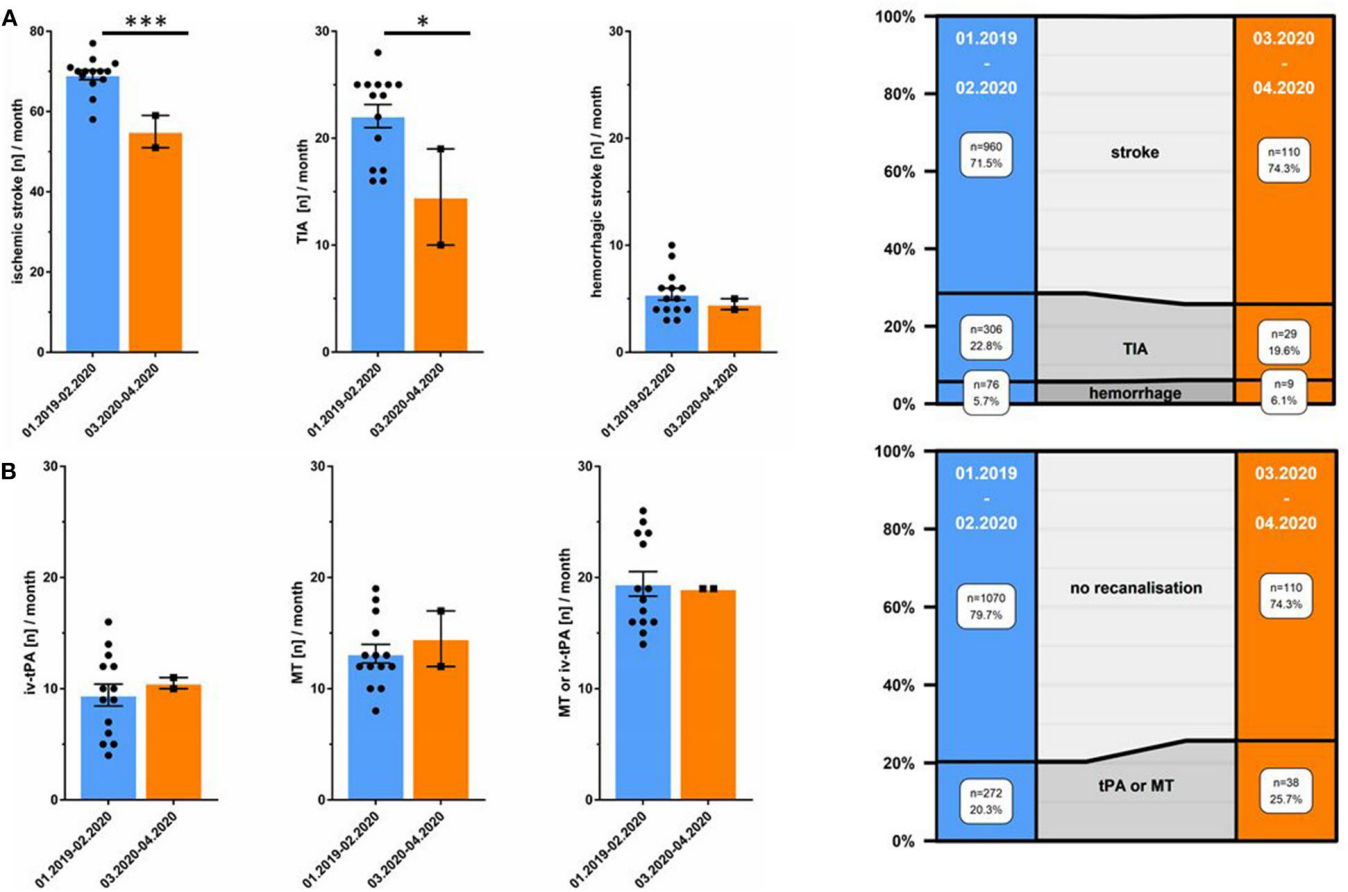
Decreased total number of ischemic stroke, hemorrhagic stroke, and TIA whereas recanalization therapies are unchanged. **(A)** Total number of patients/month in March/April 2020 (orange) and from January 2019 until February 2020 (blue). On the right: distribution of ischemic stroke, TIA, and hemorrhagic stroke between January 2019 until February 2020 as opposed to March/April 2020 during the COVID-19 pandemic. **(B)** Total number of patients/month undergoing recanalization procedure in March/April 2020 (orange) and between January 2019 until February 2020 (blue). On the right: distribution of recanalization therapies (intravenous thrombolysis [tPA] and mechanical thrombectomy [MT]) as opposed to no recanalization therapy from January 2019 until February 2020 and in March/April 2020. *** = *p* < 0.001,* = *p* < 0.05.

The percentage of patients with ischemic strokes numerically increased (*n* = 148, 74.3% in March/April 2020 vs. *n* = 960, 71.5% between January 2019–February 2020, *p* = 0.474) whereas the distribution of patients with transient symptoms (TIA) decreased (*n* = 29, 19.6% March/April 2020 vs. *n* = 306, 22.8% from January 2019–February 2020, *p* = 0.375). No relevant percentage alteration of patients with hemorrhagic stroke was observed (*n* = 9, 6.1% March/April 2020 vs. *n* = 76, 5.7% January 2019–February 2020, *p* = 0.726). The numbers of patients per month with ischemic stroke (55 ± 5.7 in March/April 2020 vs. 69.1 ± 4.5 for January 2019–February 2020, *p* < 0.001), and TIA (14.5 ± 6.4 in March/April 2020 vs. 22.1 ± 4.1 for January 2019–February 2020, *p* = 0.034) significantly decreased (see Table 1, Figure 3), whereas the number of hemorrhagic stroke patients per month remained unchanged (4.5 ± 0.7 in March/April 2020 vs. 5.5 ± 2.1 for January 2019–February 2020, *p* = 0.556). With regard to hemorrhagic stroke subtypes, we were not able to find a difference in percentages of intracerebral hemorrhage (ICH, *n* = 65, 4.8% vs. *n* = 7, 4.7%, *p* = 0.951) and subarachnoid hemorrhage (SAH, *n* = 19, 1.4% vs. 4, 2.7%, *p* = 0.277) in the non-pandemic compared to the pandemic period. Due to possible seasonal variations in the incidence of cerebral hemorrhages, we also compared percentages and number of patients per month with hemorrhagic strokes and its subtypes between March/April 2019 and March/April 2020 and observed no differences. Neither the number of patients with hemorrhagic strokes per month (*n* = 13, 6.2% vs. *n* = 9, 6.1%, *p* = 0.939), nor incidence of ICH (*n* = 12, 5.7%, vs. *n* = 7, 4.7%, *p* = 0.660) and SAH (*n* = 4, 1.9% vs. *n* = 4, 2.7%, *p* = 0.724) were different between March/April 2019 and March/April 2020 (see Table 2).

**Table 1:** Patient characteristics from January 2019 until February 2020 and in March/April 2020 during the COVID-19 pandemic.

	**01.2019–02.2020**	**03.2020–04.2020**	***p*-value**
*N*	1,342	148	
*N*, per month [Mean ± SD]	95.9 ± 6.3	74 ± 12.7	<0.001
Age [Mean ± SD]	72.6 ± 13.4	72.9 ± 12.5	0.777
Female [*n*, %]	613, 45,7%	79, 53,4%	0.075
Hospitalization [*d*]	8.2 ± 7.1	7.8 ± 6.8	0.583
Diagnosis			
Stroke, ischemic [*n*, %]	960,71.5%	110,74.3%	0.474
*N*, per month [Mean ± SD]	69.1 ± 4.5	55 ± 5.7	0.001
Stroke, hemorrhagic [*n*, %]	76,5.7%	9,6.1%	0.726
*N*, per month [Mean ± SD]	5.5 ± 2.1	4.5 ± 0.7	0.556
ICH [*n*, %]	65, 4.8%	7,4.7%	0.951
SAH [*n*, %]	19, 1.4%	4, 2.7%	0.277
TIA [*n*, %]	306,22.8%	29,19.6%	0.375
*N*, per month [Mean ± SD]	22.1 ± 4.1	14.5 ± 6.4	0.034
Treatment with t-PA [*n*, %]	132;9,5%	21,14.2%	0.098
*N*, per month [Mean ± SD]	9.4 ± 3.7	10.5 ± 0.5	0.697
Thrombectomy [*n*, %]	184,13.7%	29;19.6%	0.052
*N*, per month [Mean ± SD]	13.1 ± 3.1	14.5 ± 3.5	0.580
t-PA or thrombectomy [*n*, %]	272,20.3%	38,25.7%	0.124
*N*, per month [Mean ± SD]	19.4 ± 4.1	19.0 ± 0.0	0.889

*SD, Standard deviation; ICH, intracerebral hemorrhage; SAH, subarachnoid hemorrhage, TIA, transient ischemic attack; t-PA, tissue plasminogen activator*.

**Table 2:** Patient characteristics in non-pandemic March/April 2019 compared to March/April 2020 during the COVID-19 pandemic.

	**03/04.2019**	**03/04.2020**	***p*-value**
*N*, stroke total	207	148	
Stroke, hemorrhagic [*n*, %]	13, 6.2%	9, 6.1%	0.939
*N*, per month [Mean ± SD]	6.5 ± 3.5	4.5 ± 0.7	0.515
ICH [*n*, %]	12, 5.7%	7,4.7%	0.660
SAH [*n*, %]	4, 1.9%	4, 2.7%	0.724

*ICH, intracerebral hemorrhage; SAH, subarachnoid hemorrhage*.

With regard to recanalization therapy, we observed a trend toward a relative increase in rates of administration of iv-tPA (*n* = 21, 14.2% in March/April 2020 vs. *n* = 132, 9.5% from January 2019–February 2020, *p* = 0.098), and application of MT (*n* = 29, 19.6% March/April 2020 vs. *n* = 184, 13.7% January 2019–February 2020, *p* = 0.052). Similarly, a composite variable showed an increase in rates of iv-tPA and/or MT utilization (*n* = 38, 25.7% March/April 2020 vs. *n* = 272, 20.3% January 2019–February 2020, *p* = 0.124). Concerning the number of procedures per month, we observed no change in March/April 2020 as compared to the previous months (see Table 1, Figure 3). SARS-CoV-2 test was not mandatory; however, none of the patients included within this analysis was reported SARS-CoV-2 positive.

## Discussion

The initial weeks of the COVID-19 pandemic did not affect absolute numbers of acute stroke recanalization procedures (tPA or MT), but were found to have resulted in a decrease of the overall number of patients presenting with non-severe ischemic stroke or TIA in a primary care university hospital in Germany.

We observed a decrease by nearly one quarter in the number of patients admitted to our primary stroke center with stroke or TIA from a mean-rate of 95.9 (±6.3) per month between January 2019 and February 2020 to 74 (±12.7) in March/April 2020. Our observation is in line with numerous reports of decreased stroke rates during the pandemic from North America (4, 9, 10), Canada (5), and Brazil (6). In addition, a decrease in the usage of the RAPID-software, a tool used to assess infarct volume in case of acute stroke symptoms, was observed by mid-March 2020 (11) and telestroke services were less frequently used (9). Similar observations of reduced hospital admissions and patient presentations at emergency departments are reported in other diseases such as myocardial infarction (12, 13).

One reason for this observation might be that in March 2020 rising measures of personal and community restrictions were advised by the German government, encompassing closures of schools and numerous public places as well as prohibiting the gathering of people. Even more so, the population was advised to stay at home and avoid close contact with other individuals outside the primary family to limit the exponential spreading of COVID-19 infections. Thus, the reduced number of patients presenting with ischemic stroke and TIA observed within this analysis might be attributable to fewer people visiting the emergency department due to a fear of COVID-19 infection. This is underlined by the fact that a decrease in the percentages and absolute numbers of patients was especially detected in those presenting with transient symptoms, whereas the absolute number of recanalization therapies remained unchanged. Another explanation for decreased stroke rates might be an underestimation of stroke-related symptoms in patients with fever and respiratory symptoms. By an attempt of prioritization, neurological deficits, especially minor stroke or TIA-related symptoms, are prone to be overlooked. However, this is unlikely for disabling stroke symptoms such as aphasia and severe hemiparesis. This is in line with our observations showing that overall rates and numbers per month of recanalization procedures (iv-tPA and MT), representing patients with acute severe stroke symptoms, were unchanged in March/April 2020 compared to the previous months, and we even observed a trend toward a relative increase in percentages of recanalization procedures. Thus, we hypothesize that the absolute number of strokes is not falling during the COVID-pandemic, but patients are rather afraid of seeking hospitals and instead stay home in case of mild or transient stroke symptoms. Our hypothesis is strengthened by the fact that we observed unchanged rates of hemorrhagic stroke during the pandemic, which are usually accompanied by a more severe disability compared to ischemic strokes. This is further underlined by a retrospective analysis from Brazil also demonstrating unchanged rates of hemorrhagic stroke during the pandemic compared to pre-pandemic numbers and reporting an unchanged rate of severe strokes (6). In line with this, a multicenter analysis from Northern California reports decreased stroke rates in the early pandemic and even more severe strokes and large vessel occlusions (4).

These results underline the necessity of increasing the awareness of cerebrovascular events, in particular those with mild or transient symptoms, in order to allow for sufficient stroke unit work-up, thereby decreasing the already high rates of cerebral re-ischemia by detection of stroke etiology and consecutive prescription of secondary preventive medication (14).

The current study harbors the limitations of a retrospective, single-center experience. Moreover, a potential selection bias could exist, due to the fact that we solely included patients admitted to the neurology department stroke unit and thereby might potentially have overlooked cases, e.g., subarachnoid hemorrhages treated within the neurosurgery intensive care unit.

The overall unchanged rate of recanalization procedures in patients presenting with ischemic stroke reflects sufficient healthcare resources in a German primary stroke center during the COVID-19 pandemic. Due to a reduction in patients with minor and transient stroke symptoms, patients should be widely informed about typical stroke symptoms and encouraged not to stay at home even in exceptional situations such as a “lockdown” or a fear of in-hospital COVID-19 infection.

## Data Availability Statement

The raw data supporting the conclusions of this article will be made available by the authors, without undue reservation.

## Ethics Statement

Ethical review and approval was not required for the study on human participants in accordance with the local legislation and institutional requirements. Written informed consent for participation was not required for this study in accordance with the national legislation and the institutional requirements.

## Author Contributions

TU contributed to conception and design of the study, interpretation of data, and drafted the manuscript. SG contributed to interpretation of data and substantively revised the manuscript. EH substantively revised the manuscript. FS contributed to interpretation of data, drafted the manuscript, and substantively revised it. KG contributed to conception and design of the study, interpretation of data, and drafted the manuscript. All authors contributed to the article and approved the submitted version.

## Conflict of Interest

TU reports personal fees from Merck Serono and Pfizer, outside the submitted work. KG reports personal fees and non-financial support from Bayer AG, personal fees and non-financial support from Boehringer Ingelheim, personal fees and non-financial support from Bristol-Myers Squibb, personal fees and non-financial support from Daiichi Sankyo, personal fees and non-financial support from Pfizer, outside the submitted work. The remaining authors declare that the research was conducted in the absence of any commercial or financial relationships that could be construed as a potential conflict of interest.

## Abbreviations

COVID-19: Coronavirus Disease 2019
SARS-CoV-2: Severe acute respiratory syndrome coronavirus 2
TIA: Transient ischemic attack
ICD-10: International Statistical Classification of Diseases and Related Health Problems tenths version
iv-tPA: intravenous thrombolysis
MT: mechanical thrombectomy
IQR: interquartile range
SD: standard deviation.
